# The impact of psychosocial and organizational working conditions on the mental health of female cleaning personnel in Norway

**DOI:** 10.1186/1745-6673-1-24

**Published:** 2006-11-01

**Authors:** Migle Gamperiene, Jan F Nygård, Inger Sandanger, Morten Wærsted, Dag Bruusgaard

**Affiliations:** 1 University of Oslo, Department of General Practice and Community Medicine, Oslo, Norway; 2 University of Oslo, Akershus University Hospital, Norwegian Health Services Research Unit, Oslo, Norway; 3 The Cancer Registry of Norway, Oslo, Norway; 4 National institute of Occupational Health, Oslo, Norway

## Abstract

**Background:**

This study examined the association between psychosocial and organizational work conditions and mental health among women employed in the cleaning profession in Norway.

**Methods:**

Self-report questionnaires were mailed to 661 cleaning staff personnel from seven cleaning organizations in seven different cities across Norway. The response rate was 64%, of which 374 (88%) respondents were women. The questionnaires assessed socio-demographic information and employment history, work organization, and psychosocial working conditions. The Hopkins Symptoms Checklist (HSCL-25) was included to assess mental health.

**Results:**

On average, respondents were 43 years old and reported 10.8 years of experience working in the cleaning industry. The proportion of women scoring a HSCL-25 equal to or above 1.75 was 17.5%, which was higher than the average prevalence of mental health problems among working Norwegian women (8.4%). A factor analysis of the questions specific to the psychosocial work environment identified the following four underlying dimensions: leadership, co-workers, time pressure/control, and information/knowledge. Two of these, poor satisfaction with leadership (OR = 3.6) and poor satisfaction with co-workers (OR = 2.3), were significantly related to mental health. In addition, having contact with colleagues less than once a day (OR = 2.4) and not being ethnically Norwegian (OR = 3.0) increased the risk for mental health problems.

**Conclusion:**

Mental health problems are frequent among female cleaning professionals in Norway. Our results indicate that quality of leadership, collaboration with co-workers, and ethnicity were significantly associated with mental health.

## Background

Mental health problems impose a significant economic burden on society-at-large, employers, and individuals. The majority of the burden of mental disorders in the community arises from stress-related conditions such as anxiety and depression, collectively called the "common mental disorders" [1-4]. The financial ramifications of mental health problems at the workplace are illustrated by a US study demonstrating that depressed employees were 70% more "expensive" than their non-depressed counterparts. Employees who reported an elevated stress level which exceeded their coping abilities were 46% more costly than employees with a lower or manageable stress level. Those who reported both depression and a high stress level were 147% more "expensive" than their non-stressed, non-depressed co-workers [7]. Reports from England estimate that one-third of employees who are not able to work suffer from mental health problems, and of those, 58% are reported to be work related. In Norway, employee absenteeism due to mental disorders accounted for 16.8% of total absences and 31.5% of all refunded sick days in 1998 [5].

Worklife has been associated both with mental health problems and psychological well-being [9]. Certain working environment characteristics appear to increase an employee's susceptibility to mental health problems. It is known that in occupations with a high work pace and/or low skill discretion, the risk of mental health disorders is substantial [10]. This may explain why unskilled workers in industry and service production are reported to have a higher risk of mental health disorders compared to white-collar workers [11]. Employees in the transportation and service sector, such as health care personnel, teachers, cleaning personnel, and housekeepers are especially prone to drop out of the workforce because of mental health problems [6].

Exposure to adverse psychosocial working conditions may elevate the risk of even more severe psychiatric disorders, such as psychotic disorders. Research has shown that people in the construction trade (i.e., carpenters, painters, roofers, electricians) were 2.6 times more likely to experience delusions or hallucinations than people in managerial occupations. Furthermore, workers in housekeeping, laundry, cleaning, and servant-type occupations were 4.1 times more likely to develop schizophrenia. These associations remained stable after controlling for alcohol and drug use [12].

Nordic research suggests that a lack of job autonomy and low procedural justice (decision-making procedures) are independent risk factors for mental health problems in female employees [13]. Psychological distress may be exacerbated by the worker feeling a sense of uncontrollability and unpredictability in the work environment (e.g., corporate downsizing and reorganizing) [14]. Recent findings suggest that variables such as unfair managerial procedures and poor organizational climate result in organizational misbehaviour, lowered subjective well-being, and long sickness periods among unskilled women [15]. In contrast, the positive effects of sufficient managerial and collegial support have also been established. For example, the Whitehall II study demonstrated that social support and quality information from superiors reduced the risk for short periods of absence due to mental health problems in women, indicating directions for how to mitigate adverse trends in absenteeism [16]. Research findings generally suggest that the relationship between environmental factors and psychiatric symptoms is most prominent in women [17,18].

Cleaning is an occupation that includes many of the above-mentioned psychosocial environment characteristics associated with mental health problems. Generally, cleaning is considered to be a precarious job, with low pay, lack of esteem, lack of control over working conditions, and a lack of promotional prospects [13,25-27]. Nevertheless, most existing studies have focused narrowly on the ergonomic and chemical hazards of the cleaning profession, to the exclusion of psychosocial workplace factors [19-24].

In Norway, this occupation is characterized by a high percentage of female employees and immigrants, and a high rate of morbidity and level of disability pensioning [25]. The working environment within the cleaning profession is also characterized by a rigid structure of leadership and work organization that partly results from the absence of a permanent workplace.

Due to a relatively high turnover among employees, this occupation is difficult to investigate and thus, relatively few studies have been carried out [26,27]. The lack of knowledge regarding the psychosocial working environment and its relationship to mental health among female professional cleaning personnel provided the rationale for the present study. We aimed to explore the association between psychosocial and organizational working conditions and level of mental health distress among women employed in the cleaning profession from geographically diverse regions in Norway.

## Methods

Questionnaires were sent to 661 cleaners from seven cleaning organizations in seven different cities across Norway. The firms are organized under the NHO (Confederation of Norwegian Enterprise) and are considered to be representative of the cleaning sector as a whole. The participation rate was 64% (N = 423; 49 men and 374 women). After excluding the male respondents, 374 women comprised the final sample and were included in the analyses. The Committee for Medical Research Ethics of Norway and the Norwegian Data Inspectorate approved the study protocol.

### Dependent variable

The Hopkins Symptoms Checklist (HSCL-25) was used to assess mental health [28]. The HSCL has been found to be a psychometrically valid and reliable indicator of anxiety and depression symptomology. Anxiety and depression are common stress-related disorders and closely related to illness behaviour, such as seeking professional help, taking medication, and change in functioning [29]. Twenty-five questions, which measure the frequency and intensity of symptoms during the past week, are scored on a scale from 1 (not bothered) to 4 (extremely bothered). The HSCL-25 total score was calculated as the sum score of items divided by number of items answered. To be counted as valid and be included in the analyses, at least 13 items had to be answered. Respondents with an HSCL-25 score ≥1.75 were considered a "case" [30].

### Independent variables

The independent variables were socio-demographic data, psychosocial and work organizational characteristics. Socio-demographic data, included age and years of cleaning experience. Ethnicity was dichotomized according to whether the woman was born in Norway or not. Working time was classified according to whether the woman was working less than 37.5 hours per week (part time) or more (full time). Family status was dichotomized according to whether the woman was single or not single (married or cohabitant).

A battery of 26 questions was used to assess the psychosocial work environment over the preceding three months. The questions were selected from the General Nordic Questionnaire (QPS Nordic) [31] and included the following types of items: decision latitude, work task demand, leadership, social co-operation and competition, experience of conflicts, work challenges, and interaction between work and private life. Questions were scored on a scale from 1 (never) to 5 (almost all the time). Missing data on psychosocial work environment (4.5%–9.4%) were replaced with the mean score for the corresponding variable.

Three additional questions were used to assess work organization. These included 1) *working alone *versus *in a pair *versus *in a team*, 2) frequency of contact with colleagues, and 3) frequency of contact with managers at the workplace (*daily *versus e*very week/minimum once a month *versus *more seldom/never*).

### Statistical methods

To investigate the underlying factor structure of the 26 items on psychosocial working conditions, we conducted an exploratory factor analysis using a direct oblimin method with a non-orthogonal rotation, based on the theoretical assumption that some correlation would exist among the factors. Data considerations and statistical assumptions were met: data was quantitative at the interval scale level with a normal distribution and the sample size to item ratio was satisfactory [32].

Logistic univariate models were performed to examine the unique association between mental health and the following variables: age, cleaning experience, working time, family status, ethnicity, and dimensions of psychosocial work conditions and work organization. The final adjusted logistic multivariate regression model included only those variables that were significant predictors of mental health problems in the univariate analyses. All statistical analyses were performed with the STATA, Version 8.2.

## Results

### Demographic characteristics

The average age of the study population was 42.7 years. As shown in Table 1, 84% of all women were older than 30 years, 86.3% of the women were born in Norway, and 73.3% were married or cohabitating. Mean cleaning experience was 10.8 years, with one third (31.6%) having worked in the industry for over 15 years. Of the sample, 85.3% worked full time, 77.2% worked alone, 55.9% had daily contact with their colleagues, while 23.5% seldom or never had contact with colleagues at the workplace. Only 15.9% had daily meetings with their manager.

**Table 1:** Descriptive characteristics of N = 374 female cleaning professionals in Norway (1999)

	N	%
*Age*		
≤30	57	16.1
31–39	78	22.0
40–49	118	33.2
50–59	78	22.0
60 +	24	6.8
Missing	19	5.1
Total	355	100.0
*Work experience (years)*		
0–4	104	28.3
5–14	147	40.1
15+	116	31.6
Missing	7	1.9
Total	367	100.0
*Working time*		
Full-time	319	85.3
Part-time	55	14.7
Total	374	100.0
*Family status*		
Single	100	26.7
Not single (married/cohabitating)	274	73.3
Total	374	100.0
*Ethnicity*		
Not ethnic Norwegian	50	13.7
Ethnic Norwegian	316	86.3
Missing	8	2.1
Total	366	100.0
*Work organisational factors:*		
*Working alone/in a pair/in a team*		
Working alone	277	77.2
Working in a pair	46	12.8
Working in a team	36	10.0
Missing	15	4.0
Total	359	100.0
*Contact with colleagues at the workplace*		
Every day	205	55.9
Every week/minimum once a month	74	20.2
More seldom/never	88	24.0
Missing	7	1.2
Total	367	100.0
*Contact with manager at the workplace*		
Every day	58	15.9
Every week/minimum once a month	219	59.8
More seldom/never	89	24.3
Missing	8	2.1
Total	366	100.0

### Factor analysis

Results from the factor analysis revealed a 4-factor solution, identifying the following four psychosocial dimensions: leadership, co-workers, time pressure/control, and information/knowledge. Table 2 shows the item and factor loadings of the 26 items assessing psychosocial work characteristics. Only items loading high (>0.6) or moderately high (>0.4) were retained on a factor. For the first factor, loadings ranged from 0.4 to 0.8 and items predominantly concerned the employee-manager relationship and leadership style; thus, this factor was called "leadership." The highest loading item was "problems at work due to the lack of information from your leader" (0.8), while the lowest item loading was "you feel that the job does not fit with your ambitions" (0.4). The second factor consisted only of items about co-workers and was therefore named "co-workers." The highest item loading was "conflicts with co-workers" (0.7) and the lowest (0.4) was for the item "you experience competition with co-workers". The third factor included the items: "time pressure" and "others decide your work tempo" (.69 and .67, respectively) and this factor was named "time pressure/control". The fourth factor consisted of the items "problems at work due to the lack of information from your co-workers" (0.5), and "job demands more knowledge and experience than you can organize yourself" (0.5). This factor was named "information/knowledge". The item "others decide how you'll solve the tasks" had a clear double loading (both above 0.4) in the "leadership" and "time pressure/control" factors. Scores from each of these four factors were then divided to form three groups according to the degree of satisfaction: good, fair or poor. The resulting groups (good/fair/poor) provided the basis for examining relative risk in the logistic regression models.

**Table 2:** Factor loadings of psychosocial work conditions. Study of 374 female cleaners in Norway in 1999

	Leadership (factor 1)	Co-workers (factor 2)	Time pressure/Control (factor 3)	Information/knowledge (factor 4)
Problems at work due to the lack of information from your leader	**0.801**	-0.008	0.062	0.083
Difficult to get help from your nearest leader	**0.791**	0.120	0.097	-0.042
Leader doesn't pay enough attention to problems	**0.787**	0.092	0.042	0.046
Conflicts with leader	**0.722**	0.062	0.130	0.178
Unsure of your nearest leader	**0.703**	0.153	-0.040	-0.177
Lack of praise and encouragement at the workplace	**0.669**	0.0446	-0.095	0.146
Mistakes and problems due to the lack of education and coaching	**0.670**	0.002	0.062	0.257
You are not valued according to your efforts	**0.655**	0.008	-0.033	0.114
Poor contact with institutions' highest manager	**0.532**	-0.011	0.187	-0.117
Others decide how you'll solve the tasks	**0.503**	0.149	**0.457**	0.092
You think about problems at work in your free time	**0.483**	0.203	0.047	-0.042
You feel that the job doesn't fulfil your ambitions	**0.404**	0.049	-0.044	-0.076
Conflict with co-workers	0.107	**0.755**	0.083	-0.121
Distrust of your co-workers	0.125	**0.674**	-0.091	0.052
Co-workers don't pay enough attention when you are trying to discuss the problem	0.310	**0.640**	0.035	0.283
Collaboration with co-workers is poor	0.316	**0.595**	0.046	0.134
Difficult to get help from co-workers	0.247	**0.482**	0.083	0.258
Poor social atmosphere	0.314	**0.469**	0.011	-0.227
You experience competition among co-workers	0.117	**0.407**	0.173	-0.121
Others decide your work tempo	0.421	0.043	**0.687**	0.050
Time pressure	0.357	0.062	**0.667**	0.021
You experience competition among the managers	0.257	0.176	0.322	-0.059
Problems at work due to the lack of information from your co-workers	0.239	0.307	0.135	**0.552**
Job demands more knowledge and experience than you can organize yourself	0.343	0.080	-0.011	**0.503**
Conflicts with customer's employees	0.271	0.042	0.093	0.044
Work time creates problems for responsibilities at home	0.352	0.102	0.228	0.224

Table 3 displays the correlation matrix for the 4 dimensions of psychosocial work conditions and the three work organization variables. Results revealed no significant intercorrelations among the psychosocial work and work organization variables. The item, "meetings with colleagues at the workplace" correlated significantly with "meetings with manager at the workplace" (p ≤ .01).

**Table 3:** Correlation matrix of factors for psychosocial work conditions and work organization. Study of 374 female cleaners in Norway in 1999 (N = 352)

	Management	Co-workers	Time pressure/control	Information/Knowledge	Working alone/in a pair/in a team	Contact with colleagues at the workplace	Contact with manager at the workplace
Leadership	1.000						
Co-workers	0.039	1.000					
Time pressure/control	0.064	0.011	1.000				
Information/knowledge	0.031	0.042	0.001	1.000			
Working alone/in a pair/in a team	0.038	-0.057	0.109	0.048	1.000		
Contact with colleagues at the workplace	0.013	-0.058	-0.088	-0.011	-0.219	1.000	
Contact with manager at the workplace	0.119	-0.200	-0.057	-0.097	-0.226	0.464*	1.000

* P = 0.0002

### HSCL-25

A total of 354 women completed the HSCL-25 questions. The mean score was 1.41, with 17.5% (62 of 354) reporting an HSCL-25 score ≥1.75. Of those with elevated scores, the mean was 2.16 (CI 2.06 – 2.25). The two groups did not differ significantly in age (mean ages were 42.5 and 43.4 years, respectively) or experience (10.6 and 11.6 years, respectively).

### Univariate logistic regression

Table 4 shows the crude odds ratios for the univariate associations between the independent variables (socio-demographic, psychosocial work dimensions, and work organization) and the risk of having an elevated HSCL-25 score. Results demonstrated that fair and poor satisfaction with leadership had a significant association with mental health problems (OR = 2.6 and 3.8, respectively). Specifically, the cleaners who were least satisfied with their leadership had a significantly higher mean HSCL score than women who were satisfied (1.56 and 1.25, respectively; not shown in the table). Poor satisfaction with co-workers also had a significant association with mental health problems (OR = 2.0). Specifically, the mean HSCL score was higher among women who were least satisfied with co-workers than women who were satisfied (1.52 and 1.41 respectively; not shown in the table). Compared with meeting colleagues every day, meeting colleagues at the workplace every week/minimum once a month or seldom/never appeared to be related to mental health problems (OR = 2.5 and 1.9, respectively). Those cleaners who met their colleagues every week/minimum once a month had a significantly higher HSCL score than women who met their colleagues every day (1.53 and 1.35 respectively; not shown in the table).

**Table 4:** Logistic univariate relationship between mental health and personal, work organization, and psychosocial work environment variables among female cleaners in Norway in 1999

	HSCL≥1.75		
Risk factors	N	OR	95% CI
	
Age (p = 0.6)	340		
≤30 (ref.)		1.0	-
31–39		0.9	0.4 – 2.5
40–49		1.1	0.5 – 2.6
50–59		1.6	0.7 – 4.0
60+		0.8	0.2 – 3.2
			
Work experience (years) (p = 0.8)	350		
0–4 (ref.)		1.0	
5–14		1.2	0.6 – 2.4
15+		1.1	0.5 – 2.3
			
Working time (p = 0.3)	354		
Full time (ref.)		1.0	
Part time		1.5	0.7 – 3.1
			
Family status (p = 0.2)	354		
Single (ref.)		1.0	
Not single (married/cohabitating)		0.7	0.4 – 1.3
			
Ethnicity (p < 0.01)	351		
Ethnic Norwegian (ref.)		1.0	
Not ethnic Norwegian		2.8	1.4 – 5.5
			
Psychosocial risk factors (from factor analysis):			
Satisfaction with leadership (model p < 0.001)	354		
Good (ref.)		1.0	
Fair		2.6	1.2 – 5.8
Poor		3.8	1.8 – 8.1
			
Satisfaction with co-workers (model p = 0.01)	354		
Good (ref.)		1.0	
Fair		0.7	0.4 – 1.6
Poor		2.0	1.1 – 3.9
			
Satisfaction with time pressure/control (model p = 0.3)	354		
Good (ref.)		1.0	
Fair		0.8	0.4 – 1.6
Poor		1.3	0.6 – 2.6
			
Satisfaction with information/knowledge (model p = 0.01)	354		
Good (ref.)		1.0	
Fair		0.3	0.2 – 0.7
Poor		0.8	0.4 – 1.5
			
Work organisational risk factors:			
Working alone/in a pair/in a team (model p = 0.3)	344		
Working alone (ref.)		1.0	
Working in a pair		0.8	0.3 – 1.8
Working in a team		0.4	0.1 – 1.4
			
Contact with colleagues at the workplace (model p = 0.02)	352		
Every day (ref.)		1.0	
Every week/min once a month		2.5	1.3 – 4.8
More seldom/never		1.9	1.0 – 3.7
			
Contact with manager at the workplace (model p = 0.6)	351		
Every day (ref.)		1.0	
Every week/min once a month		1.5	0.6 – 3.6
More seldom/never		1.6	0.6 – 4.1

Working alone rather than in a pair or team had no significant association with mental health problems, nor did the frequency of employee meetings with the manager. Working part time represented a higher, but not significant, risk of an elevated HSCL-25 score. Ethnicity, however, was significantly related to mental health problems. Those who were not ethnic Norwegians had a significantly greater risk of mental health problems than ethnic Norwegians (OR = 2.8; mean HSCL-25 scores were 1.62 and 1.37, respectively). No significant association was found between the HSCL-25 and the following variables: age, years of cleaning experience, or family status.

### Multiple logistic regression

We included the following variables in the adjusted multivariate logistic regression model: age, ethnicity, satisfaction with leadership, co-workers, information/knowledge, and meeting colleagues at the workplace (see Table 5). Women aged 50–59 years had a higher risk of mental health problems than other age groups (OR = 3.2). Other variables demonstrating a significant association with mental health problems included: fair and poor leadership, poor satisfaction with co-workers, meeting colleagues less than every day, and ethnicity.

**Table 5:** Logistic multivariate regression analyses of mental health according to age, working time, ethnicity, work organization, and psychosocial work environment variables among female cleaners in Norway in 1999 (N = 351)

	HSCL≥1.75	
Risk factors	OR	95% CI
		
Age		
≤30 (ref.)	1.0	
31–39	1.2	0.4 – 3.4
40–49	2.0	0.8 – 5.3
50–59	3.2	1.2 – 8.5
60+	2.1	0.5 – 9.4
		
Ethnicity		
Ethnic Norwegian (ref.)	1.0	
Not ethnic Norwegian	3.0	1.4 – 6.4
		
Psychosocial risk factors (from factor analysis):		
Satisfaction with leadership		
Good (ref.)	1.0	
Fair	2.2	1.8 – 6.2
Poor	3.6	1.2 – 10.6
		
Satisfaction with co-workers		
Good (ref.)	1.0	
Fair	1.6	0.6 – 4.1
Poor	2.3	1.1 – 4.8
		
Satisfaction with information/knowledge		
Good (ref.)	1.0	
Fair	0.7	0.3 – 1.7
Poor	0.8	0.4 – 1.6
		
Work organisational risk factors:		
Contact with colleagues at the workplace		
Every day (ref.)	1.0	
Every week/min once a month	2.4	1.2 – 5.1
More seldom/never	2.0	0.9 – 4.1

## Discussion

Our study investigated the association between psychosocial and organizational work conditions and mental health among female cleaning personnel in Norway. Approximately eighteen percent (17.5%) of our sample reported mental health problems. Results illustrated several key distinguishing psychosocial, organizational, and demographic characteristics, which significantly influenced mental health. Cleaning personnel reporting a poor relationship with their leader or colleagues were more likely to have elevated symptoms of anxiety and depression. Similarly, cleaning staff who were not ethnically Norwegian had a greater risk of mental health problems.

In our study we utilized a data collected in a self-report manner via a cross-sectional survey. All data were therefore dependent upon the employee's momentary psychological state and subject to biases associated with self-report. Both burnout and depression can effect the perception or experience of work stressors [33]. Some studies have shown that *subjective *appraisal of work conditions correlates more strongly with self-reported depression than *objective *work conditions [34]. Moreover, it has been argued that the relation between work stress and depression may simply be attributable to underlying career frustration [27,35], which was not addressed in the current study. It is important to note that the pathways linking psychosocial work conditions and mental health may not be direct, but reciprocal and bidirectional. Thus, it cannot be precluded that the cleaners' mental state affected the report of psychosocial work conditions and work organization.

Our study focused on the mental health and its relationship to psychosocial working conditions for women. Cleaning is predominantly a female occupation. In the European Union (EU), it is estimated that private enterprises, governments, and local authorities employ nearly three million full- and part-time cleaners, 95% of whom are women [38]. Owing to observed gender differences in psychological distress and the higher propensity of women to report mental health problems associated with the psychosocial work environment than men [18,39], we chose to exclude men from this study. However, future research investigating mental health issues among male cleaning professionals represents an interesting area of study.

The HSCL-25 was chosen as the primary index of mental health distress in the present study. Although less comprehensive in scope than a structured interview, the HSCL-25 has been psychometrically established in both population studies and in patient populations [40] and imposes minimal time and resource demands upon participants. It has also shown a high agreement with physicians' ratings of emotional distress [41] and is considered to be a satisfactory indicator of mental health. The chosen cut-off of 1.75 is identical to standards used in previous workplace and population studies [18,30,40,42], permitting direct comparison of the results to other studies.

Although a handful of studies have reported high levels of morbidity and disability among cleaning staff [26,43,44], a host of methodological challenges such as high turnover and part-time employment have limited research activity within this field. Our study included female cleaning personnel from geographically diverse regions in Norway. Moreover, participants were employed in well-organized firms of various sizes. As the majority of respondents were working full time, more than 80% were older than 30 years, and one-third had more than 15 years of experience, our sample may reflect a rather stable fraction of women employed in the cleaning profession. Thus, our findings may provide more favourable results for working conditions and mental health than can be expected in the cleaning sector as a whole.

In our sample of female cleaning staff, the proportion of women scoring HSCL-25 above or equal to 1.75 was 17.5%, which is higher than results from a national survey which found an 8.4% prevalence level of mental health problems among average working Norwegian women [42]. At least two explanatory mechanisms may exist to account for this observation. First, the work environment itself may have led to the development of mental health problems. However, a prior study found that the risk of obtaining a disability pension among cleaning staff did not increase with a longer duration of work experience [25]. Second, it could be argued that our findings are attributable to a selection effect, whereby women with mental health problems are more likely to enter the cleaning profession–i.e., an unhealthy worker effect. Such a negative selection might result in an over-estimated health risk within the cleaning occupation.

A majority of our items assessing the psychosocial working environment reflected the quality of the relationship between the employee and her manager and colleagues. The factor analysis revealed four meaningful psychosocial work dimensions, and these included leadership, co-workers, time pressure/control, and information. Results from the univariate analyses showed an association between mental health and poor leadership, as well as between mental health and unsatisfactory collaboration with colleagues. These results are consistent with results from a Swedish population study, which demonstrated similar findings for other types of professions [45,46]. In addition, infrequent contact with colleagues (less than everyday) was also associated with mental health problems. Such dissatisfaction with the quality of social contacts has been associated with an increased risk for impaired psychological well-being in women, and thus has been introduced as an independent predictor of distress [33,47]. Differential evidence, however, has been supported to specify the prognostic value of social support for mental health. One study found an effect only for those who had specific and multiple work stressors [48]. In a community sample [49], only the support of a supervisor reduced the risk of depression over one year, while support from a colleague did not. Conflicting views therefore remain whether social support operates as an independent risk factor for morbidity, or simply moderates the relationship between stressors and psychological morbidity; to date, the evidence more strongly supports the former [50].

The two factors, "time pressure/control" and "information/knowledge," were not significantly associated with mental health problems. Surprisingly, no associations were found between working time, work organization, and mental health problems. Our findings are inconsistent with results from previous studies, in which occupational factors such as shift work and job strain were related to poor mental health among women [46,47].

Regarding demographic characteristics, we found that cleaning personnel aged 50–59 years had the highest prevalence of mental health problems. This age trend is consistent with findings from a national survey of working women in Norway [42]. An earlier study, investigating the risk of disability pensioning among cleaning staff members, found an even higher risk for disability pensioning in this age group [25]. Work – family conflicts and striking a balance between these two important areas of life has been found to impact the mental health of women in many industrialized countries [54]. In contrast, our item, "Work time creates problems for responsibilities at home," failed to load on the four factors and similarly, family status showed no significant association with mental health. Research has found that both work and non-work stressors contribute to level of depression [55], but these issues were beyond the scope of the present study.

We found that being an immigrant was a significant risk factor for mental health problems among female cleaning staff in Norway. Cultural norms and sanctions operate at the national, local, and individual level, which undoubtedly influence women's roles both in the household and workplace. Studies on migration have shown that the stress of adaptation and settlement, as well as language barriers, may negatively affect a person's mental health and contribute to the development of depression [51]. In a study involving a multi-ethnic population, the relationship between ethnicity and mental health was found to be associated with socio-economic status (SES) [52]. The authors concluded that depression associated with a low socio-economic status might arise from adverse psychosocial conditions at work [53]. Results of our study provide some support for these conclusions.

## Conclusion

Mental health problems were common among female cleaning personnel in Norway. Our results indicated that mental health was associated with the quality of leadership and collaboration with co-workers, as well as with ethnicity. High quality collaboration between the cleaning staff and their leaders appears to be more important than the quantity of meetings. We emphasize the importance of frequent on-the-job social contact and good collegial relationships for women working in the cleaning profession.
